# Optimal chemo-mobilization for the collection of peripheral blood stem cells in patients with multiple myeloma

**DOI:** 10.1186/s12885-019-5285-1

**Published:** 2019-01-14

**Authors:** Ga-Young Song, Sung-Hoon Jung, Seo-Yeon Ahn, Seung-Yeon Jung, Deok-Hwan Yang, Jae-Sook Ahn, Hyeoung-Joon Kim, Je-Jung Lee

**Affiliations:** Department of Hematology-Oncology, Chonnam National University Hwasun Hospital, 322 Seoyangro, Hwasun, Jeollanamdo 519-763 Republic of Korea

**Keywords:** Stem cell mobilization, Multiple myeloma, Etoposide

## Abstract

**Background:**

For successful autologous stem cell transplantation, the collection of a sufficient number of hematopoietic stem cells after induction therapy is essential for transplant candidates with multiple myeloma (MM).

**Methods:**

In this study, we compared the efficacy and safety of stem cell mobilization using cyclophosphamide (CY; 3.0 g/m^2^ on day 1) or etoposide (VP-16; 375 mg/m^2^ on days 1 and 2) in patients with MM. Granulocyte-colony stimulating factor (G-CSF, 10 μg/kg/day, subcutaneously) was administered from the onset of neutropenia to the final day of collection.

**Results:**

Sixty-five patients were mobilized with a combination of CY and G-CSF, and 63 were mobilized with a combination of VP-16 and G-CSF. All patients were mobilized within 7 months of beginning frontline treatment. The median number of CD34^+^ cells collected was significantly higher in the VP-16 mobilization group than in the CY mobilization group (27.6 ×  10^6^ CD34^+^/kg vs. 9.6 × 10^6^ CD34^+^/kg, *P* <  0.001). The rate of mobilization failure, defined as < 2.0 × 10^6^ CD34^+^/kg collected in three apheresis procedures, was lower in the VP-16 group than in the CY group (1.6% vs. 10.8%, *P* = 0.062). Severe infections during the mobilization period were more frequent in the CY group than in the VP-16 group (18.5% vs. 7.9%, *P* = 0.117).

**Conclusion:**

In conclusion, an intermediate dose of VP-16 with G-CSF appears to be an effective and tolerable chemo-mobilization method compared to CY and G-CSF, particularly in cases where use plerixafor in MM is difficult.

## Background

With the emergence of new and more effective therapeutic agents, the role of autologous stem cell transplantation (ASCT) in multiple myeloma (MM) has become a topic of considerable interest. Although there have been only a few studies evaluating the efficacy of ASCT with novel agent-based therapies, upfront ASCT did show a better response and a longer progression free survival (PFS) in a randomized phase 3 EMN02/HO95 trial (HR = 0.76; 95% confidence interval [CI] = 0.64–0.91; *P* = 0.002). Furthermore, a recently published meta-analysis of three large phase 3 randomized controlled trials showed the superiority of upfront ASCT over novel agents [1, 2]. Delaying ASCT until a relapse after frontline novel agent-based therapy is also accepted as an alternative treatment option [3, 4]. Therefore, high-dose chemotherapy combined with ASCT continues to be a well-established treatment strategy for transplant-eligible MM patients.

In MM patients, peripheral blood stem cells (PBSCs) are the most common cell source for ASCT [5]. As obtaining a sufficient dose of stem cells is important to achieve stem cell engraftment, numerous strategies have been developed to expand the pool of circulating hematopoietic stem cells [6]. The standard method to mobilize stem cells is to treat them with granulocyte colony stimulating factor (G-CSF), with or without cyclophosphamide (CY) [7, 8]. Chemo-mobilization achieves higher yields of stem cells than growth factor alone but requires more time for the engraftment of platelets and neutrophils, and carries an increased risk of infection [9]. Introduction of CY improves the efficacy of mobilization, but still results in a high rate of mobilization failure (10~20%) [10]. Therefore, more effective chemo-mobilization protocols are needed.

VP-16 is effective in mobilizing hematopoietic stem cells in patients with malignant lymphoma [11, 12]. Wood et al. [13] demonstrated that mobilization with 375 mg/m^2^ of VP-16 and G-CSF was highly effective and safe in MM patients. In their study, all patients achieved successful mobilization with a median number of CD34^+^ cells of 12 × 10^6^/kg. Moreover, there were no reported collection failures, and no treatment-related mortalities. However, no studies have compared CY and VP-16 chemo-mobilization in MM patients. Therefore, we evaluated the efficacy and safety of stem cell mobilization using intermediate doses of VP-16 and high doses of CY in patients with MM.

## Methods

### Patients

We retrospectively analyzed the medical records of 140 patients diagnosed with MM who underwent stem cell mobilization between February 2008 and February 2018 at Chonnam National University Hwasun Hospital. We excluded patients who mobilized with G-CSF alone. Patients who were mobilized more than 12 months after initial treatment for delayed ASCT were also excluded. Following these exclusions, a total of 128 patients were included in this study. This study was approved by the Institutional Review Board of Chonnam National University Hwasun Hospital in accordance with the Declaration of Helsinki.

### PBSC mobilization and collection

Stem cells were mobilized with G-CSF following intravenous CY (3.0 g/m^2^ on day 1) or VP-16 (375 mg/m^2^ on days 1 and 2) administration. G-CSF (10 μg/kg/day, subcutaneously) was administered from the onset of neutropenia (absolute neutrophil count < 1 × 10^9^/μL) up to the final collection day. For all patients, antimicrobial and antifungal prophylaxis was given using oral levofloxacin 500 mg/day and fluconazole 200 mg/day during the neutropenic period. Daily monitoring of circulating hematopoietic progenitor cells (HPCs), enumerated by the Sysmex XE2100 (Sysmex Corporation, Kobe, Kansai, Japan), was initiated at the start of leukocyte recovery, approximately 8 days after chemotherapy. Apheresis was initiated when HPC levels reached ≥5/μL, as described previously [14].

### Definition

The International Staging System (ISS) was used to assess the clinical disease stage at diagnosis [15]. The International Myeloma Working Group uniform response criteria were used to assess the response to treatment [16]. Toxicity was graded according to the National Cancer Institute Common Terminology Criteria for Adverse Events (v4.0). Mobilization failure was defined as the total number of collected CD34^+^ cells < 2.0 × 10^6^ CD34^+^/kg. Platelet engraftment after transplant was defined as the first of 7 consecutive days where the platelet count was > 20 × 10^9^/L without platelet transfusion. Neutrophil engraftment after transplant was defined as the first of 3 consecutive days where the absolute neutrophil count was > 0.5 × 10^9^/L without the administration of G-CSF. The duration of hospitalization was defined as the number of days from the initiation of the conditioning regimen for ASCT to the day of discharge.

## Statistical analyses

Discrete and continuous variables were evaluated using the chi-square test and a Mann-Whitney *U*-test, respectively. The PFS was calculated from the day of transplantation to disease progression or death from any cause. Overall survival (OS) was calculated from the day of transplantation to the day of death from any cause. PFS and OS were evaluated using the Kaplan-Meier method, and compared using the log-rank test. Estimates of the relative risk for an event and its 95% CI were obtained using the Cox proportional hazard model. All statistical analyses were performed using SPSS software (ver. 21.0; SPSS Inc., Chicago, IL, USA). A *P* value < 0.05 was considered statistically significant.

## Results

### Patients

Between 2008 and 2018, a total of 128 patients with MM underwent stem cell mobilization and collection using CY or VP-16 within 12 months of their initial diagnosis. Sixty-five patients (50.8%) were mobilized with CY + G-CSF between February 2008 and December 2013, and 63 (49.2%) were mobilized with VP-16 + G-CSF between April 2014 and February 2018. The baseline clinical characteristics of both groups are summarized in Table 1. Overall, the patients had similar disease characteristics such as ISS stage, immunoglobulin subtype, serum levels of lactate dehydrogenase (LDH) and creatinine, baseline levels of hemoglobin, and platelet counts. Patients had received a median of one (range, 1–3) prior treatment regimen before stem cell mobilization. Nine patients (13.8%) in the CY mobilization group received two prior treatment regimens, whereas all except one patient in the VP-16 mobilization group received one treatment regimen. The frontline treatment differed slightly between the two groups, with more patients being treated with a bortezomib-containing regimen in the VP-16 group (65.1% vs. 23.1%, *P* <  0.001). Despite differences in frontline treatment, disease responses to the initial regimen before stem cell mobilization were similar in the two groups (*P* = 0.359). The median time from the first treatment to mobilization was shorter in the VP-16 group than the CY group (3.88 months vs. 4.67 months, *P* <  0.001) however, there were no differences in the median number of treatment cycles prior to mobilization (4 vs. 4, *P* = 0.056). The number of patients who received spinal radiotherapy was similar in both groups (27.7% vs. 23.8%, *P* = 0.616).

**Table 1:** Baseline clinical characteristics of patients with different chemo-mobilization regimens (*n* = 128)

Variables	Cyclophosphamide (*n* = 65)	Etoposide (*n* = 63)	*P*-value
Median age, year (range)	55.0 (39 ~ 64)	57.0 (34 ~ 65)	0.359
Male, *n* (%)	39 (60.0%)	36 (57.1%)	0.743
Ig type, *n* (%)			0.173
IgG	35 (53.8%)	44 (69.8%)	
IgA	8 (12.3%)	6 (9.5%)	
IgM	1 (1.5%)	1 (1.6%)	
Light chain only	21 (32.3%)	11 (17.5%)	
ISS, *n* (%)			0.356
I	22 (33.8%)	15 (23.8%)	
II	21 (32.3%)	27 (42.9%)	
III	22 (33.8%)	21 (33.3%)	
ECOG PS ≥ 2, *n* (%)	6 (9.2%)	2 (3.2%)	0.274
LDH > (1 × ULN), *n* (%)	7 (10.9%)	11 (17.5%)	0.292
Median BM plasma cells, %, range	35.0 (4.0 ~ 90.0)	27.0 (0.9 ~ 95.8)	0.068
Median lymphocyte count, (× 10^9^/L), range	2.00 (0.4 ~ 6.23)	2.19 (0.57 ~ 4.78)	0.340
Serum creatinine ≥2 mg/dL, n (%)	13 (20.3%)	8 (12.7%)	0.340
Median platelet count (× 10^9^/L), range	201 (48 ~ 393)	206 (63 ~ 509)	0.802
Median serum Hb, g/dL	9.8 (4.9 ~ 15.3)	10.0 (6.2 ~ 15.4)	0.577
Median time to mobilization, months (range)	4.67 (2.53 ~ 6.80)	3.88 (2.43 ~ 6.18)	< 0.001
Frontline treatment, n (%)			
Thalidomide-based	59 (81.9%)	20 (31.3%)	
Bortezomib-based	7 (9.7%)	14 (21.9%)	
Bortezomib+Thalidomide	0 (0.0%)	29 (45.3%)	
Conventional chemotherapy	6 (8.3%)	1 (1.6%)	
Median prior treatment cycle, *n*, range	4 (3 ~ 7)	4 (3 ~ 6)	0.056
Radiotherapy (spine), *n* (%)	18 (27.7%)	15 (23.8%)	0.616
Disease status at mobilization			0.359
CR	12 (18.5%)	16 (25.4%)	
VGPR	14 (21.5%)	7 (11.1%)	
PR	36 (55.4%)	38 (60.3%)	
SD	3 (4.6%)	2 (3.2%)	
PD	0 (0.0%)	0 (0.0%)	

Abbreviations: *N* number, *ISS* international staging system, *ECOG* Eastern Cooperative Oncology Group, *PS* performance status, *LDH* lactate dehydrogenase, *ULN* upper limit of normal value, *BM* bone marrow, *Hb* hemoglobin, *CR* complete response, *VGPR* very good partial response, *PR* partial response, *SD* stable disease, *PD* progressive disease

### Efficacy and survival outcome

**Fig. 1:**
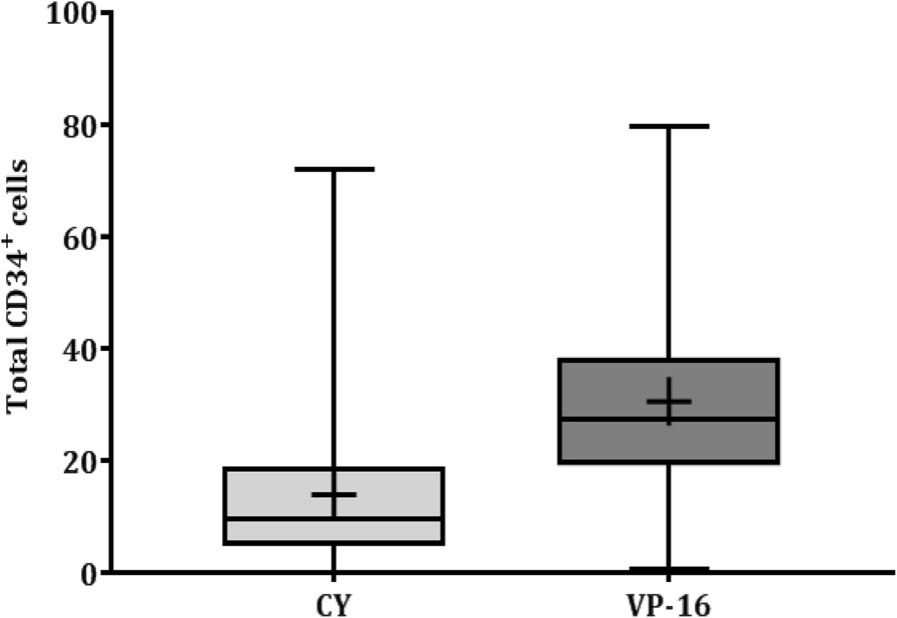
Total number of CD34^+^ cells collected (× 10^6^ CD34^+^/kg) according to the different collection regimens

The mobilization data are summarized in Table 2. Fewer patients failed stem cell collection in the VP-16 mobilization group than in the CY mobilization group (1.6% vs. 10.8%, *P* = 0.062). In the VP-16 group, only one patient showed mobilization failure and also failed to provide a sufficient amount of stem cells in a subsequent mobilization attempt using plerixafor. Among the seven patients who had mobilization failure with CY, three decided not to pursue ASCT, one received a bone marrow harvest, one underwent a repeated mobilization procedure with CY, and two received a second mobilization procedure with G-CSF only. The median number of days for leukapheresis was significantly shorter in the VP-16 group than in the CY group (2 days vs. 3 days, *P* <  0.001). The median number of CD34^+^ cells collected on day 1 was significantly higher in the VP-16 group than in the CY group (12.6 × 10^6^ CD34^+^/kg vs. 2.8 × 10^6^ CD34^+^/kg, *P* <  0.001, Fig. 1). In patients who received radiotherapy, the number of CD34^+^ cells collected was significantly higher in the VP-16 group than in the CY group (24.5 × 10^6^ CD34^+^/kg vs. 8.68 × 10^6^ CD34^+^/kg, *P* <  0.001). As a result, patients were transplanted with a larger number of hematopoietic stem cells in the VP-16 group than in the CY group (16.15 × 10^6^ CD34^+^/kg vs. 5.71 × 10^6^ CD34^+^/kg, *P* <  0.001).

**Table 2:** Mobilization data

	Cyclophosphamide (*n* = 65)	Etoposide (*n* = 63)	*P* value
Median days of leukapheresis, days (range)	3.0 (2–5)	2.0 (2–3)	< 0.001
Median number of CD34^+^ cells (× 10^6^ CD34^+^/kg)	9.6 (0.2–71.9)	27.6 (0.6–79.8)	< 0.001
Median number of CD34^+^ cells collected at day 1 (× 10^6^ CD34^+^/kg)	2.8 (0.1–31.4)	12.6 (0.5–39.9)	< 0.001
Patients with yield < 2.0 × 10^6^ CD34^+^/kg, *n* (%)	7 (10.8)	1 (1.6)	0.062
Patients with yield < 4.0 × 10^6^ CD34^+^/kg, *n* (%)	12 (18.5)	1 (1.6)	< 0.001

Fifty-seven patients (87.7%) in the CY group and 56 (88.9%) in the VP-16 group underwent upfront ASCT. The outcomes following ASCT are summarized in Table 3. Most patients in the CY group received a high-dose melphalan conditioning regimen (84.2%), whereas VP-16 group patients received either a melphalan and other agent (48.2%) or a thiotepa and busulfan (35.7%) conditioning regimen. The median time to platelet engraftment was longer in the VP-16 group than in the CY group (12 days vs. 9 days, *P* = 0.055). The median number of patients requiring platelet transfusion support during the transplantation period was fewer in the VP-16 group (2 vs. 4, *P* <  0.001). There were no significant differences between the two groups with respect to the median time to neutrophil engraftment. The duration of post-PBSC infusion hospitalization was shorter in the VP-16 group.

**Table 3:** Result of subsequent autologous stem cell transplantation

	Cyclophosphamide (*n* = 57)	Etoposide (*n* = 56)	*P* value
Conditioning regimen, *n* (%)			< 0.001
High-dose melphalan	48 (84.2)	9 (16.1)	
Melphalan with another agent	9 (15.8)	27 (48.2)	
Busulfan-Thiotepa	0 (0.0)	20 (35.7)	
Median infused CD34^+^ cells (× 10^6^ CD34^+^/kg)	5.71 (1.85 ~ 12.60)	16.15 (7.10 ~ 39.00)	< 0.001
Post PBSC infusion hospitalization duration, days (range)	15 (13~65)	14 (12~54)	0.005
Time to neutrophil engraftment (> 0.5 × 10^9^/L), days	11 (8 ~ 17)	10 (8 ~ 15)	0.292
Time to platelet engraftment (> 20 × 10^9^/L), days	9 (6 ~ 32)	12 (7 ~ 13)	0.055
Median number of platelet transfusions support during transplantation (range)	4 (1~12)	2 (0~8)	< 0.001
Pre-engraftment complications, *n* (%)			
Neutropenic fever	41 (73.2)	36 (67.9)	0.674
Hepatic veno-occlusive disease	0 (0.0)	0 (0.0)	
Hemorrhagic cystitis	0 (0.0)	0 (0.0)	
Engraftment syndrome	6 (11.1)	7 (12.5)	1.000
Early graft failure	0 (0.0)	0 (0.0)	
Treatment-related mortality	1 (1.8)	2 (3.6)	0.618

**Fig. 2:**
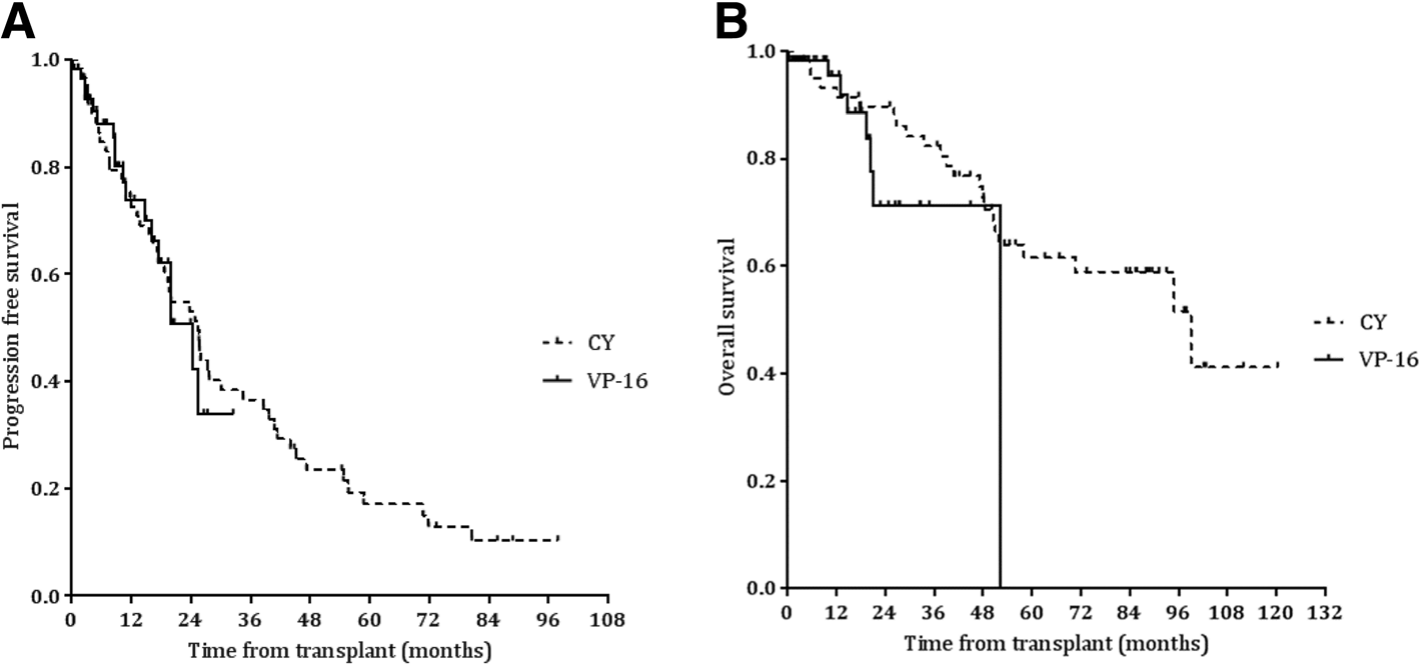
Kaplan-Meier survival curves for progression free survival (**a**) and overall survival (**b**) in all patients

After a median follow-up time of 84.0 months in the CY group and 12.6 months in the VP-16 group, PFS and OS were not significantly different between the groups (PFS, 25.4 vs. 24.3 months, *P* = 0.806, 99.1 months vs. not reached, *P* = 0.113, Fig. 2a, b).

### Safety

More patients in the CY group required supportive transfusion during the mobilization period, which is defined as the time between the first day of mobilization chemotherapy infusion and the last day of collection. In all, 42 (64.6%) patients in the CY group and 25 (39.7%) in the VP-16 group needed transfusion with red blood cells (*P* = 0.008). In addition, 47 (72.3%) patients in the CY group and 24 (38.1%) in the VP-16 group required platelet transfusions (*P* < 0.001). Non-hematological toxicities occurred in 35.4% of the CY group and 22.2% of the VP-16 group. Following mobilization chemotherapy, the most common non-hematological toxicity was infection. The overall incidence of infection after mobilization was higher in the CY group (32.3% vs. 22.2%, *P* = 0.237). Severe infection (≥ grade 3) also occurred more often in the CY group; however, this difference was not statistically significant (18.5% vs. 7.9%, *P* = 0.117). All patients who developed severe infections were successfully treated with broad spectrum antibiotics, which resulted in no patient deaths in either group. Other non-hematological toxicities included gastrointestinal and hepatotoxicity. One patient in the CY group, and one in the VP-16 group, showed a grade 2 aspartate transaminase/alanine transaminase elevation, which recovered following supportive care. One patient showed a mild hypersensitive reaction, including pruritus and facial swelling, after infusion of CY, which was resolved by chlorpheniramine injection. Two patients complained of grade 1 or 2 nausea and vomiting, and were also relieved of these symptoms after supportive care. There was no treatment-related mortality during the mobilization period in either patient group.

## Discussion

There is little consensus on the most appropriate regimen for stem cell mobilization for MM. The addition of plerixafor, a chemokine receptor type 4 inhibitor, to G-CSF has shown good mobilization results in many studies and has ignited the pursuit to improve mobilization regimens [17–19]. However, there is a lack of research examining both the long-term efficacy and adverse effects of plerixafor. In addition, it is not clearly defined when plerixafor should be used as a primary mobilization regimen, nor what type of patients should be targeted. Another important factor is that plerixafor is expensive and many countries do not subsidize its use, which may impose a financial burden on many patients. As conventional chemo-mobilization is still expected to play a large role in ASCT, alternatives to plerixafor are highly desirable. Improved mobilization using VP-16 is meaningful in this sense, as it is a promising candidate as a plerixafor replacement.

In patients mobilized with VP-16, many more stem cells could be collected safely over fewer days of apheresis compared to CY mobilization. The number of patients for whom an adequate amount of stem cells could not be collected was also lower in the VP-16 group. The time from the first treatment to mobilization was also longer in the CY mobilization group than the VP-16 mobilization group (4.67 months vs. 3.88 months, *P* < 0.001). We surmise that these differences are due to disparities in the induction regimen schedule between the two groups. The majority of induction regimens at our institution follow a 28-day schedule; however, some regimens have a 21-day schedule. A greater number of patients in the VP-16 mobilization group were treated using the 21-day schedule regimen, although the number of induction cycles prior to mobilization were not different between the two groups. As a result, the median time to mobilization was slightly longer in the CY group than in the VP-16 group. A previous study on VP-16 mobilization in MM demonstrated that VP-16 successfully mobilized an adequate amount of stem cells (≥ 5.0 × 10^6^ CD34^+^/kg) in patients who were previously given radiation therapy [13]. An evaluation of the subgroup of patients who received prior radiotherapy showed that the stem cell yield was higher in VP-16 patients than in CY patients. Although there were no significant differences in patients who failed mobilization, the number of CD34^+^ cells collected was significantly different between the two methods. Although the number of patients evaluated was too small to warrant definitive conclusions, we speculate that the use of VP-16 is able to overcome the adverse effects of previous radiotherapy.

In this study, the incidence of infection was higher in the CY group than in the VP-16 group. Because of its highly immunosuppressive effect, CY has been used for the treatment of autoimmune diseases and acute or chronic graft-versus-host disease after allogenic hematopoietic stem cell transplantation. A significant decrease in the percentage of both central and effector memory T cells has also been reported in a murine model treated with CY [20]. In a related experiment, VP-16 selectively eliminated activated T cells, but did not deplete either quiescent naïve or memory T cells [21]. Although the exact etiology is unknown, we speculate that CY has a more profound immunosuppressive effect than VP-16, which increases the incidence of infection.

This study also included a patient in whom a sufficient number of stem cells failed to be collected after VP-16 mobilization. The subsequent use of plerixafor for immediate salvage was also unsuccessful. Plerixafor-containing regimens have around a 20% failure rate when implemented after failed mobilization, most likely as a result of a low or defective stem cell reserve [18, 22]. Factors influencing successful stem cell mobilization include age, prior bone marrow radiotherapy, prior exposure to alkylating agents, and the number of previous chemotherapy cycles [23–26]. Bone marrow reserve, as characterized by platelet count, or marrow cellularity prior to mobilization, may also have an effect on the quality of stem cell mobilization [27, 28]. Therefore, more research examining stem cell reserves will be necessary to enhance the efficacy of mobilization and to prevent unnecessary suffering.

Because this study was retrospective in nature, the conditioning regimens in both groups differed significantly, and these differences might have influenced the transplantation results, such as time to neutrophil or platelet engraftment, and survival outcomes.

## Conclusion

In conclusion, the median number of CD34^+^ cells collected from patients who were mobilized with an intermediate dose of VP-16 and G-CSF was significantly higher compared to those patients who were mobilized with CY and G-CSF. The mobilization failure rate was also lower in the VP-16 mobilization group than in the CY mobilization group. Although there were no statistically significant differences, severe infections during the mobilization period developed more frequently in the CY mobilization group. Therefore, an intermediate dose of VP-16 and G-CSF might be a more suitable chemo-mobilization regimen than a regimen of CY and G-CSF, particularly in situations where plerixafor cannot be implemented.

## Abbreviations

ASCT: Autologous stem cell transplantation
BM: Bone marrow
CI: Confidence interval
CR: Complete response
CY: Cyclophosphamide
ECOG: Eastern Cooperative Oncology Group
G-CSF: Granulocyte-colony stimulating factor
Hb: Hemoglobin
HPCs: Hematopoietic progenitor cells
ISS: International Staging System
LDH: Lactate dehydrogenase
MM: Multiple myeloma
N: number
OS: Overall survival
PBSCs: Peripheral blood stem cells
PD: Progressive disease
PFS: Progression free survival
PR: Partial response
PS: Performance status
SD: Stable disease
ULN: Upper limit of normal value
VGPR: Very good partial response
VP-16: Etoposide

## References

[1] Cavo M, Hájek R, Pantani L, Beksac M, Oliva S, Dozza L, Johnsen HE, Petrucci MT, Mellqvist U-H, Conticello C, Driessen C, Marzocchi G, Dimopoulos MA, Zweegman S, Wu KL, Gamberi B, Crippa C, van der Holt B, Offidani M, Wester R, Vincelli ID, Troia R, Cornelisse P, Boccadoro M, Sonneveld P. Autologous stem cell transplantation versus Bortezomib-Melphalan-prednisone for newly diagnosed multiple myeloma: second interim analysis of the phase 3 EMN02/HO95 study. Blood. 2017;130(Suppl 1) 397–397.

[2] Dhakal B, Szabo A, Chhabra S, Hamadani M, D'Souza A, Usmani SZ, Sieracki R, Gyawali B, Jackson JL, Asimakopoulos F, Hari PN (2018). Autologous transplantation for newly diagnosed multiple myeloma in the era of novel agent induction: a systematic review and meta-analysis. JAMA Oncology.

[3] Attal M, Lauwers-Cances V, Hulin C, Leleu X, Caillot D, Escoffre M, Arnulf B, Macro M, Belhadj K, Garderet L, Roussel M, Payen C, Mathiot C, Fermand JP, Meuleman N, Rollet S, Maglio ME, Zeytoonjian AA, Weller EA, Munshi N, Anderson KC, Richardson PG, Facon T, Avet-Loiseau H, Harousseau J-L, Moreau P (2017). Lenalidomide, Bortezomib, and dexamethasone with transplantation for myeloma. N Engl J Med.

[4] Dunavin NC, Wei L, Elder P, Phillips GS, Benson DM, Hofmeister CC, Penza S, Greenfield C, Rose KS, Rieser G, Merritt L, Ketcham J, Heerema N, Byrd JC, Devine SM, Efebera YA (2013). Early versus delayed autologous stem cell transplant in patients receiving novel therapies for multiple myeloma. Leuk Lymphoma.

[5] Körbling M, Freireich EJ (2011). Twenty-five years of peripheral blood stem cell transplantation. Blood.

[6] Tricot G, Jagannath S, Vesole D, Nelson J, Tindle S, Miller L, Cheson B, Crowley J, Barlogie B (1995). Peripheral blood stem cell transplants for multiple myeloma: identification of favorable variables for rapid engraftment in 225 patients. Blood.

[7] Chitra H, QM H, Partow K, Sergio G, DM S, Uday P, Paolo A, SE J, John M, Martin K, CR E (2006). Fixed-dose single agent pegfilgrastim for peripheral blood progenitor cell mobilisation in patients with multiple myeloma. Br J Haematol.

[8] Awan F, Kochuparambil ST, Falconer DE, Cumpston A, Leadmon S, Watkins K, DeRemer D, Jillella A, Craig M, Hamadani M (2013). Comparable efficacy and lower cost of PBSC mobilization with intermediate-dose cyclophosphamide and G-CSF compared with plerixafor and G-CSF in patients with multiple myeloma treated with novel therapies. Bone Marrow Transplant.

[9] Gertz MA, Kumar SK, Lacy MQ, Dispenzieri A, Hayman SR, Buadi FK, Dingli D, Gastineau DA, Winters JL, Litzow MR (2009). Comparison of high-dose CY and growth factor with growth factor alone for mobilization of stem cells for transplantation in patients with multiple myeloma. Bone Marrow Transplant.

[10] Giralt S, Costa L, Schriber J, DiPersio J, Maziarz R, McCarty J, Shaughnessy P, Snyder E, Bensinger W, Copelan E, Hosing C, Negrin R, Petersen FB, Rondelli D, Soiffer R, Leather H, Pazzalia A, Devine S (2014). Optimizing autologous stem cell mobilization strategies to improve patient outcomes: consensus guidelines and recommendations. Biol Blood Marrow Transplant.

[11] Reiser M, Josting A, Draube A, Mapara MY, Scheid C, Chemnitz J, Tesch H, Wolf J, Diehl V, Söhngen D, Engert A (1999). Successful peripheral blood stem cell mobilization with etoposide (VP-16) in patients with relapsed or resistant lymphoma who failed cyclophosphamide mobilization. Bone Marrow Transplant.

[12] Wood WA, Whitley J, Goyal R, Sharf A, Irons R, Rao KV, Essenmacher A, Serody JS, Coghill JM, Armistead PM, Sarantopoulos S, Gabriel DA, Shea TC, Brown P (2013). Effectiveness of etoposide Chemomobilization in lymphoma patients undergoing autologous stem cell transplantation. Bone Marrow Transplant.

[13] Wood WA, Whitley J, Moore D, Sharf A, Irons R, Rao K, Serody J, Coghill J, Gabriel D, Shea T (2011). Chemomobilization with etoposide is highly effective in patients with multiple myeloma and overcomes the effects of age and prior therapy. Biol Blood Marrow Transplant.

[14] Jung S-H, Park H, Ahn J-S, Yang D-H, Kim M-Y, Kim Y-K, Kim H-J, Lee J-J (2013). Efficacy of stem cell mobilization in patients with newly diagnosed multiple myeloma after a CTD (cyclophosphamide, thalidomide, and dexamethasone) regimen. Int J Hematol.

[15] Greipp PR, Miguel JS, Durie BGM, Crowley JJ, Barlogie B, Bladé J, Boccadoro M, Child JA, Avet-Loiseau H, Kyle RA, Lahuerta JJ, Ludwig H, Morgan G, Powles R, Shimizu K, Shustik C, Sonneveld P, Tosi P, Turesson I, Westin J (2005). International staging system for multiple myeloma. J Clin Oncol.

[16] Kumar S, Paiva B, Anderson KC, Durie B, Landgren O, Moreau P, Munshi N, Lonial S, Bladé J, Mateos M-V, Dimopoulos M, Kastritis E, Boccadoro M, Orlowski R, Goldschmidt H, Spencer A, Hou J, Chng WJ, Usmani SZ, Zamagni E, Shimizu K, Jagannath S, Johnsen HE, Terpos E, Reiman A, Kyle RA, Sonneveld P, Richardson PG, McCarthy P, Ludwig H, Chen W, Cavo M, Harousseau J-L, Lentzsch S, Hillengass J, Palumbo A, Orfao A, Rajkumar SV, Miguel JS, Avet-Loiseau H (2016). International myeloma working group consensus criteria for response and minimal residual disease assessment in multiple myeloma. Lancet Oncol.

[17] DiPersio JF, Stadtmauer EA, Nademanee A, Micallef INM, Stiff PJ, Kaufman JL, Maziarz RT, Hosing C, Früehauf S, Horwitz M, Cooper D, Bridger G, Plerixafor CG (2009). G-CSF versus placebo and G-CSF to mobilize hematopoietic stem cells for autologous stem cell transplantation in patients with multiple myeloma. Blood.

[18] Duarte RF, Shaw BE, Marín P, Kottaridis P, Ortiz M, Morante C, Delgado J, Gayoso J, Goterriz R, Martínez-Chamorro C, Mateos-Mazón JJ, Ramírez C, de la Rubia J, Achtereekte H, Gandhi PJ, Douglas KW, Russell NH (2010). Plerixafor plus granulocyte CSF can mobilize hematopoietic stem cells from multiple myeloma and lymphoma patients failing previous mobilization attempts: EU compassionate use data. Bone Marrow Transplant.

[19] Dugan MJ, Maziarz RT, Bensinger WI, Nademanee A, Liesveld J, Badel K, Dehner C, Gibney C, Bridger G, Calandra G. Safety and preliminary efficacy of plerixafor (Mozobil) in combination with chemotherapy and G-CSF: an open-label, multicenter, exploratory trial in patients with multiple myeloma and non-Hodgkin's lymphoma undergoing stem cell mobilization. Bone Marrow Transplant 2009; 45:39. 10.1038/bmt.2009.119 19483760

[20] Włodarczyk M, Ograczyk E, Kowalewicz-Kulbat M, Druszczyńska M, Rudnicka W, Fol M. Effect of cyclophosphamide treatment on central and effector memory T cells in mice. Int J Toxicol. 2018;1091581818780128. 10.1177/1091581818780128 29923437

[21] Johnson TS, Terrell CE, Millen SH, Katz JD, Hildeman DA, Jordan MB (2014). Etoposide selectively ablates activated T cells to control the immunoregulatory disorder hemophagocytic lymphohistiocytosis. J Immunol (Baltimore Md : 1950).

[22] García-Escobar I, Parrilla L, Ortega LM, Castellanos D, Pallarés MA, Cortés-Funés H (2014). Clinical experience with plerixafor as a mobilization regimen for autologous peripheral blood stem cell transplantation in patients with refractory germ cell tumors. Mol Clin Oncol.

[23] Nicolas K, Gilles S, Isabelle M, Charles D, Assia EC, Pierre T, Catherine T, Brigitte D, Eve-Marie NB, Hanadi S, Dominique R, Bertrand C (1998). Factors associated with successful mobilization of peripheral blood progenitor cells in 200 patients with lymphoid malignancies. Br J Haematol.

[24] Kobbe G, Söhngen D, Bauser U, Schneider P, Germing U, Thiele KP, Rieth C, Hünerlitürkoglu A, Fischer J, Frick M, Wernet P, Aul C, Heyll A (1999). Factors influencing G-CSF-mediated mobilization of hematopoietic progenitor cells during steady-state hematopoiesis in patients with malignant lymphoma and multiple myeloma. Ann Hematol.

[25] de la Rubia J, Blade J, Lahuerta J, Ribera J, Martinez R, Alegre A, Garcia-Larana J, Fernandez P, Sureda A, de Arriba F, Carrera D, Besalduch J, Garcia Boyero R, Palomera Bernal L, Hernandez M, Garcia P, Perez-Calvo J, Alcala A, Casado L, San Miguel J (2006). Effect of chemotherapy with alkylating agents on the yield of CD34+ cells in patients with multiple myeloma. Results of the Spanish myeloma group (GEM) study. Haematologica.

[26] Stiff PJ (1999). Management strategies for the hard-to-mobilize patient. Bone Marrow Transplant.

[27] Kuittinen T, Nousiainen T, Halonen P, Mahlamäki E, Jantunen E (2004). Prediction of mobilisation failure in patients with non-Hodgkin's lymphoma. Bone Marrow Transplant.

[28] Hosing C, Saliba RM, Ahlawat S, Körbling M, Kebriaei P, Alousi A, De Lima M, Okoroji J-G, McMannis J, Qazilbash M, Anderlini P, Giralt S, Champlin RE, Khouri I, Popat U (2009). Poor hematopoietic stem cell mobilizers: a single institution study of incidence and risk factors in patients with recurrent or relapsed lymphoma. Am J Hematol.

